# 3D Printing of Layered Structures of Metal-Ionic Polymers: Recent Progress, Challenges and Opportunities

**DOI:** 10.3390/ma16155327

**Published:** 2023-07-28

**Authors:** Angelo Martinelli, Andrea Nitti, Riccardo Po, Dario Pasini

**Affiliations:** 1 Department of Chemistry, INSTM Research Unit, University of Pavia, Via Torquato Taramelli 12, 27100 Pavia, Italy; 2 Energies, Renewable Energies and Materials Science Research Center, Donegani Institute, Eni Spa, Via Giacomo Fauser 4, 28100 Novara, Italy

**Keywords:** ionic polymer-metal composites, 3D printing, additive manufacturing

## Abstract

Layered Structures of Metal Ionic Polymers, or Ionic Polymer-Metal Composites (IPMCs) are formed by a membrane of an ionic electroactive materials flanked by two metal electrodes on both surfaces; they are devices able to change their shape upon application of an electrical external stimulus. This class of materials is used in various fields such as biomedicine, soft robotics, and sensor technology because of their favorable properties (light weight, biocompatibility, fast response to stimulus and good flexibility). With additive manufacturing, actuators can be customized and tailored to specific applications, allowing for the optimization of performance, size, and weight, thus reducing costs and time of fabrication and enhancing functionality and efficiency in various applications. In this review, we present an overview of the newest trend in using different 3D printing techniques to produce electrically responsive IPMC devices.

## 1. Introduction

Additive manufacturing (AM) based on 3D printing technologies is considered one of twelve potential disruptive technologies that will lead to the next industrial revolution by 2025, and which therefore have a tremendous impact on economy and society in general [1,2]. Initially used as a prototyping platform to create objects using model data from computer aided design (CAD) drawings, 3D printing technologies are nowadays used for the production of end-user products as a consequence of the technological improvements obtained in terms of quality, price and manufacturing process times. AM technologies are currently used extensively in the aerospace, automotive/motor racing, machine tool production, healthcare and biomedical, construction, jewelry, fashion, food and entertainment fields [3,4].

Three main 3D printing technologies are marketed that differ from each other according to manufacturing process: (a) extrusion-based processes (solid feedstock materials such as filament or pellets); (b) photopolymerization processes (liquid feedstock materials) and (c) powder bed fusion processes (powder feedstock materials). In the extrusion-based processes, the molding materials are present as a filament, which is heated in the printing head of the 3D printer to obtain a viscoelastic melt, which then flows in a highly viscous manner as it is forced out through the nozzle of the extruder. Fused deposition modeling (FDM) is the most widely utilized extrusion-based technology, it is simple to set up but presents a low resolution (30–120 μm) [5,6]. Photopolymerization AM technologies involve the curing of a photoactive resin using a light source to create prototypes, models, and patterns through a layer-by-layer approach. Cost effectiveness, flexibility, and precision (the resolution of these methodologies is close to 7 μm) are the main benefits of these technologies, of which stereolithography (SLA) or direct laser printing (DLP) are the most utilized methodologies. The materials utilized in these procedures are various and also determine their versatility in multi-material 3D printing [7]. In powder bed fusion (PBF) technologies, instead, microscopic particles of powder are melted or bound layer by layer to construct 3D objects. The heat source used can vary from a pulsed laser to an electron beam or even UV light. Polymers, metals, ceramics, and composites are among the materials used in this technique. This technology has found widespread use in the AM of metallic parts for aerospace and biomedical applications despite its relatively high costs and low achievable resolution (85 μm).

The growing success of AM technologies can be ascribed to increasingly precise and fast printing techniques, but also to the development of printable materials and composites which confer the desired properties to objects. The materials that can presently be manufactured utilizing 3D printing technology include traditional thermoplastics, metals, graphene-based materials, electroactive polymers and ceramics. Electroactive polymers (EAPs) are a class of materials that have gained significant attention in the field of electronics and robotics due to their unique properties. These polymers possess the ability to change their shape, size, and mechanical properties in response to electrical stimuli, making them ideal for actuation and sensing purposes. EAPs are made up of long-chain polymers that exhibit electroactive behavior as result of the presence of mobile ions or polarizable groups within the polymer structure which, under an external electric field, undergo reorientation leading to a change in the shape or globular size of the polymer. This responsiveness to electrical stimuli allows EAPs to function as soft actuators [8,9], artificial muscles and skins [10,11], electromechanical sensors [12], robotic components [13], and micro-valves/pumps [14].

**Figure 1:**
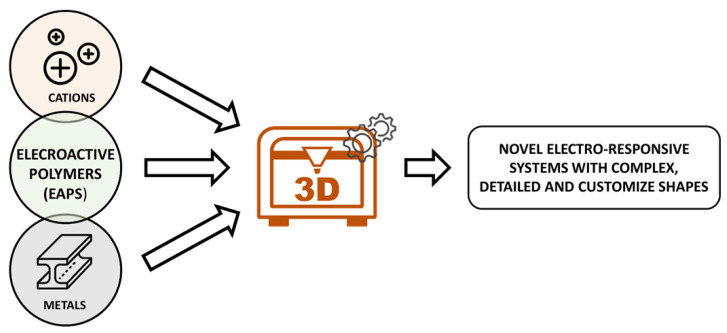
Schematic construction of electroactive IPMCs described this review.

There are various types of EAPs, each with their own unique properties and applications. One such type is the Layered Structures of Metal Ionic Polymers (IPMCs), which consist of thin ion-exchange polymer membranes sandwiched between two metal electrodes. IPMCs can undergo significant bending or actuation in response to a low voltage due to the migration of ions within the polymer (Figure 1). The best advantage in the use of IPMCs consists of an electro-induced mechanical displacement induced by low electrical voltages (<5 V). Due to ionic migration, IPMCs can bend in opposite directions without the use of specially designed structures thanks to the polarity of the applied voltage. Conducting polymers (CP) [15] and carbon nanotubes (CNT) [16] have been used as actuators in IPMCs [17].

The use of non-conventional procedures such as additive manufacturing (AM) techniques in the design of IPMCs is a less explored area compared to the additive manufacturing of others EAPs such conductive polymers [18] or poly(ionic liquid)s [19], but these procedures can be employed to fabricate complex and novel electro-responsive systems with detailed shapes and architectures. This review article provides an overview of the recent developments in IPMCs processed using AM technologies. The review will first describe the relevant AM methodologies and their features, and then it will briefly discuss IPMCs and, for each 3D printing technique, provide relevant cases of IPMC 3D printing.

## 2. Results and Discussion

This section is divided in two parts. In the first part of the review (Section 2.1), the basic concepts of several 3D printing techniques and fundamental descriptions of the relevant devices are reported. Researchers that have familiarity with the fundamental parameters used to control 3D printing could jump directly to the second part of dissertation (Section 2.2). In the second part, we describe the mechanism of actuation and the elementary steps for actuator fabrication and provide an analysis of the literature on the use of 3D printing techniques in design fabrications. The last part of the section should provide a concise and precise description of the experimental results and their interpretation, as well as the experimental conclusions that can be drawn.

### 2.1. An Overview on the 3D Printing Methodologies Used for Fabrication of IPMCs

The 3D printing process starts with the design of a CAD model which is then divided into 2D layers by a slicing program and sent to a machine to create the desired object layer by layer. This technology offers a wide range of benefits, including faster production times, more intricate designs and reduced waste compared to traditional manufacturing methods, and the opportunity to work with different materials such as plastics [20], conductive polymers [18], metals [21], ceramics [22], or a combination of them to create multi-material objects.

**Figure 2:**
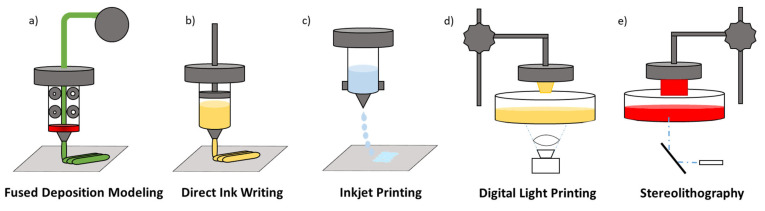
Top: (**a**–**e**) Schematic representations of FDM, DIW, IP, SLA and DLP techniques. Bottom: principal highlights and weakness for each technique discussed.

3D printing techniques for polymers can be broadly grouped into two macro-categories: (1) material extrusion-based technologies that include fused deposition modeling (FDM), direct ink writing (DIW), inkjet printing (IP) and similar methods, which build up the object by heating and extruding a polymer filament or ink (Figure 2a); and (2) photopolymerization-based technologies such as SLA and DLP, as shown in Figure 2b, which build up the object by the curing of a liquid-photoactive resin using light sources (laser, LED or a projector) [23]. Each of these two categories has its own set of advantages and disadvantages, and the choice of which one to use will depend on the specific requirements of the project [24].

#### 2.1.1. Fused Deposition Modeling (FDM)

This methodology works by heating a thermoplastic polymer filament to a semi-solid (melt) state and extruding it through a small, heated nozzle to build up a three-dimensional object layer by layer. As the melted polymer is extruded from the nozzle, it solidifies and adheres to the previous layer of material, forming the walls and structure of the 3D object. The process is repeated layer by layer until the entire object is complete. For a complete description of the technologies, we recommended reading references [25,26]. In this subsection, we highlight the important concepts used in Section 2.2 of this work.

During the process, it is important control the temperature and nozzle diameter because both parameters affect the quality (a non-optimal temperature causes filament degradation), resolution, and time of printing (an optimal nozzle size in the range of 0.2–0.8 mm affects the surface quality and mechanical properties of the finished product). Also, extrusion speed can be changed to optimize time and product quality; in fact, higher speeds result in faster printing times but poor-quality manufacturing. Finally, the filament–print platform interaction parameters are important for obtaining an object with good quality. Adhesion to the printing plate can be improved by using a heated build platform (0 °C to 80 °C), using an adhesion promoter, or using a surface with a texture that promotes adhesion.

The commonly used polymers are ABS (Acrylonitrile Butadiene Styrene), PLA (Polylactic Acid), PVA (Polyvinyl Alcohol), Nylon, TPE (Thermoplastic Elastomers) and PC (Polycaprolactone) [27]. A high fluidity index is needed, so that the polymers can melt easily and flow smoothly through the nozzle without clogging, together with a good thermal stability to avoid degradation processes during printing.

#### 2.1.2. Direct Ink Writing (DIW)

In direct ink writing (DIW), the 3D printing process is similar to the previously discussed FDM, but instead of melting and extruding thermoplastic filaments, DIW uses a nozzle to deposit a stream of ink or paste, composed of a polymer (ABS, PLA or PCL [36] PVA [37], PEDOT:PSS [38], PANI [39] or lignin [40]) suspended or solubilized in a solvent, onto a substrate. After ink deposition, solidification occurs either naturally or is assisted by an external process such as solvent evaporation, gelation, solvent-driven reactions, heat treatment, or photocuring [28]. The DIW process does not require high temperatures to extrude the material and it is possible to work at room temperature, opening up the possibility to use a wide range of thermolabile materials.

Many of the previously described parameters also influence DIW. To be able to obtain well-defined objects, the ink must flow smoothly, even at lower extrusion pressures, without discontinuity or particle jamming that can otherwise clog the deposition nozzle. This behavior is controlled by the shear-thinning parameters of the materials, that is, the non-Newtonian behavior of fluids whose viscosity decreases with increasing shear rate; typically, the viscosity of the DIW inks falls between the 10^2^ mPa·s and 10^6^ mPa·s range at a shear rate of approximately 0.1 s^−1^ to ensure printability of the inks. At the end of the deposition process, the printed structure must retain its shape until fully solidified, and it is necessary to prevent the stacked filaments from sagging under their own weight. The loss tangent (tan δ) is the critical parameter that describes this behavior and is defined as the ratio between the loss modulus (*G*″) and storage modulus (*G*′). Its value must be less than 1, and to obtain good performance in printing in general, the *G*′ value of inks must remain nearly constant over low shear stress.

These parameters are of paramount importance for the successful completion of any DIW process, both in terms of printability and shape fidelity [29,30].

#### 2.1.3. Inkjet Printing (IP)

Inkjet printing is a type of digital printing technology that involves using a thermal, piezoelectric, or electromagnetic stimulus to generate and then spray droplets of ink onto a surface to create an image or text [14,15]. Inkjet printing is a versatile digital printing technology that is used in a wide variety of applications, including wearable electronics [33], sensors [41], and biomedical engineering [42].

According to the mechanism of droplet generation, there are two main types of inkjet printing [33]. The continuous inkjet printing (CIJ) process involves continuously propelling ink droplets from a nozzle onto a surface, regardless of whether or not they are needed to create the image. This method is often used for high-speed printing of variable data, such as barcodes or serial numbers. Drop-on-demand inkjet printing (DOD) involves spraying ink droplets onto the surface only when they are needed to create the image. In this technique, an electric current heats up a tiny resistor, causing a small amount of ink to vaporize and form a droplet that is then propelled onto the surface. Low-boiling-point solvents such as water or short-chain alcohols are required [43].

There are some physical properties of ink that play fundamental roles in droplet formation, jetting, and deposition processes. The ink should have a low viscosity (typically between 1–25 mPa s), and the surface tension should be low (between 20–50 mN·m^−1^), which helps the ink droplets to form a uniform shape and spread out evenly on the printing surface. For a better and reproducible printability, the ink should be stable over time, having minimal changes in viscosity, surface tension, and dispersion uniformity in the solvent or carrier fluid, and it should dry relatively quickly after printing to prevent smudging or smearing of the image.

#### 2.1.4. Stereolithographic 3D Printing (SLA)

Stereolithography (SLA) is a 3D printing process that uses a laser to cure a liquid resin into a solid part. During the printing process, a build platform is lowered into the vat where a liquid photoresin is placed, mainly consisting of monomers (like acrylates, methacrylates, epoxides and vinyl compounds) and a photoinitiator [44,45], and subsequently, a laser is used to trace the first layer of the model onto the surface of the resin point by point. Wherever the laser light hits the resin, it causes a chemical reaction that solidifies the resin. The platform is then lowered by the thickness of one layer, and the laser traces the second layer, which adheres to the first. This process is repeated layer by layer until the entire part is complete. Once the 3D printed object is fully cured, it is removed from the vat and rinsed in a solvent bath to remove any excess resin. The part may also be post-cured in a UV oven to improve its mechanical properties and increase its durability [21,22].

SLA is used in both the ceramic [46] and polymeric materials fields [47] for its ability to produce highly accurate and detailed parts with very smooth surface finishes [48,49]. One potential drawback of SLA is that it can be relatively slow. Additionally, the resin used in SLA can be expensive, which may limit its use for some applications.

#### 2.1.5. Digital Light Processing (DLP)

DLP is quite similar to SLA. The 3D object grows layer by layer on an elevator plate by curing a liquid photoresin, but in this case, the light source is a projector or another visual display device. It is thus possible to project the entire slice where the photopolymerization takes place, which greatly shortens the printing process when compared to SLA [3]. High-quality objects with a higher resolution (very smooth surface) can be obtained [50,51,52,53,54].

### 2.2. Ionic Polymer-Metal Composites Devices (IPMCs)

**Figure 3:**
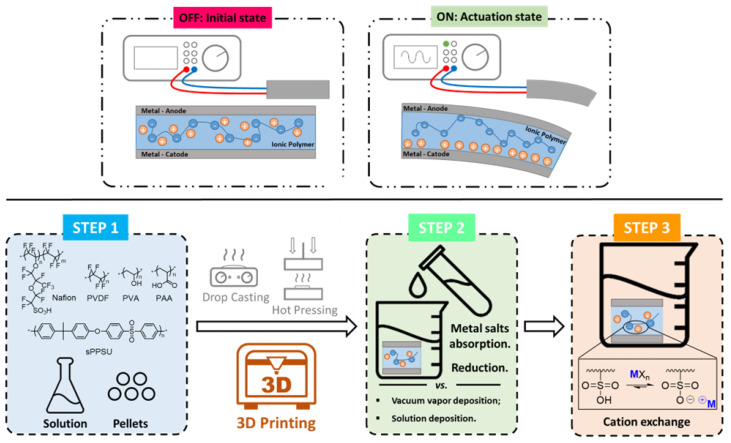
Schematic representation of three steps involved in the fabrication of IPMC devices.

IPMCs are actuator devices formed by ionic-electroactive polymer membranes sandwiched between two electrodes deposited by chemical or physical methods. Upon external electrical stimulus, actuation occurs through shape changes of the membrane/electrode system along its major or minor axis, depending on the shape of the membrane. The middle layer of ion-conductive polymers is responsible for the actuations acting as a channel for the selective movement of cations that interact with an external electric field, whereas the external metallic electrodes allow the transmission of external electric field information to the polymeric membrane (Figure 3). This sandwich structure enables a fast response to electrical stimulus [55], large bending displacement [56], good stability and high force [57]. Thanks to these properties, IPMCs are used in different fields like soft robotics, flexible sensors, biomedicine and for biomimetic applications [58].

The mechanism of actuation involves the application of a voltage to the electrodes and an electric field is generated between the two layers (Figure 3 top). The electric field promotes the movement of the free cations within the central anion membrane toward the cathode, and the negative charges will remain stationary as they are part of the polymeric framework. This migration causes expansion of the cathode and contraction of the anode, which macroscopically results in bending or general deformation of the IPMC.

Device fabrication requires the three fundamental steps described in Figure 3 (bottom): (a) membrane fabrication (step 1 in the blue box); (b) electrode depositions on both the membrane surfaces (step 2 in the green box); and (c) final doping with selected metal counterions (step 3 in the orange box).

Nafion^®^ [56], eventually combined with other polymers [59], is the most widely used polymer for membrane fabrication due to its commercial availability, chemical stability and the ability of the sulfonate groups to exchange protons with metallic counterions [60]. Other polymers such as polyvinylidene fluoride (PVDF) [61,62], polyvinyl alcohol (PVA), sulfonated polyphenylene sulfone (sPPSU) [63,64], and polyacrylic acid (PAA) have been reported [65]. Once the appropriate polymer for the IPMC membrane is chosen, it undergoes processing to attain the desired thickness and shape. Two primary methods are employed: (1) solution casting and (2) hot pressing. The solution casting technique involves pouring a polymer solution into a mold with the desired shape, allowing it to dry, and repeating the process until the desired thickness is achieved. In the hot-pressing process, films are stacked and subjected to high temperature and pressure, resulting in a single layer of the desired thickness. These methods have been joined in recent years by a third method of preparation, 3D printing, the topic of this review. Some advantages/disadvantages of the three preparation methods are summarized in Table 1.

In the second step of device fabrication, the ionic membranes are roughened and noble metals (Pt, Pd, Ag, Au or Ni) are deposited to form the two thin-layer electrodes [55,70,71]. More cost effective and easy solution depositions have also been used for the preparation of IPMCs using platinum-copper alloy [72], self-healing alloy [73], or conductive materials as carbon nanotubes (CNT) [74,75], graphene [76], or conductive polymers such as PEDOT:PSS [77]. The most used methods of deposition are carried out by using direct or reverse electroless plating preparations, which have the advantage of providing durable IPMCs with a greater life cycle. During direct electroless plating, also known as impregnation-reduction, the polymer membrane is immersed in a metal salt solution to be impregnated with the metal and the samples are subsequently immerged in a solution with a reducing agent to reduce the metal cations into metal particles. This process is repeated until the target thickness of the metal electrodes is achieved [78]. The reverse electroless plating method involves first, having the reducing agent absorbed into the ionic membrane, and then having the artifact immersed in a solution of metal salts to promote the redox reaction. This process leads to greater penetration of metal particles and thus a thicker electrode layer [79,80].

Finally, in the third step, an exchange reaction between the H^+^ of sulfonic groups and metals such as Li^+^, K^+^, Na^+^, Ca^2+^, Mg^2+^, Ba^2+^ is carried out by diffusion of the corresponding salts from a carrier solution [81].

Traditional methods for preparing IPMC membranes consist of coating commercially available sheets [77] or tubes [78] of Nafion or other polymers with platinum or other noble metals. The use of predefined starting shapes limits the applicable geometries and consequently affects the applicability of IPMCs. The use of 3D printing techniques for membrane preparation have overcome this obstacle thus opening up the technology to a host of new applications. In the follow-up discussion, we report on the main recent contributions related to 3D printing techniques for the preparation of IPMC actuators.

#### 2.2.1. IPMC Devices from FDM Methodology

**Figure 4:**
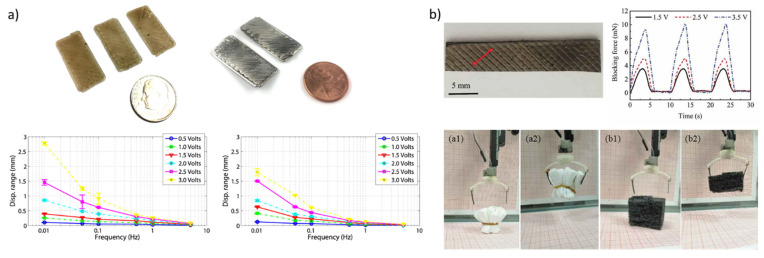
Nafion-based IPMC printed by FDM. Adapted from refs. [82,83]. (**a**) (top left): printing of the membrane and coating with two layers of platinum; (bottom left): comparison of the bending performance of the 3D printed membrane (left) with the control tester; (**b**) (top right): Cu^2+^ ion IPMC with a Nafion membrane; (bottom right): the flexible grippers made by two IPMCs: (**a1**,**b1**) the initial state of the gripper; (**a2**,**b2**) the state of grasping the object.

In 2015, J. D. Carrico and coworkers reported, for the first time, the use of FFF 3D printing to create a Nafion membrane-based IPMC. To overcome the unmeltability of Nafion, the authors converted the commercially available pellets precursor, the NAFION^®^ R1100, into a 1.75 mm diameter filament, which was extruded at a temperature of 280 °C, with a rate of 30 mm·s^−1^ on a printer plate at 80 °C. The membrane was printed with a wavy pattern diagonal to its length, and then it was hydrolytically treated with a 15 wt% KOH/35 wt% DMSO/50 wt% distilled water solution. After 4 h, the membrane was completely hydrolyzed and was coated with two layers of platinum (Figure 4a). The bending performance of the membrane exhibited a maximum displacement of 0.57 mm in response to a 10 mHz (after the application of four successive stimuli), 2.5 V amplitude that was slightly higher (0.51 mm) than the control tester made from a commercially available Nafion membrane, but the relaxation time was twice as long as that of the control (50 s vs. 24 s) [82].

Very recently, the creation of a new Cu^2+^ ion IPMC with a Nafion membrane printed by setting the nozzle and the bed temperature at 280 °C and 120 °C, respectively, and using a printing speed about 20 mm·s^−1^ (Figure 4b) was reported. The printed Nafion membrane showed higher capacity of ion-exchange (IEC) when compared with commercial Nafion-115 or Nafion-117 membranes (0.81 meq·g^−1^ vs. 0.71 meq·g^−1^), resulting in a higher maximum operating current (0.30 A), displacement (7.57 mm) and blocking force (10.5 mN, that reached a plateau of 5 mN over 300 s) under a square wave input of 3.5 V and 0.1 Hz, over three tests [83]. In follow-up studies, the same group, using the same membrane type, reported the fabrication of new patterns and new forms of actuators [84,85,86].

Nafion is not the only perfluorinated polymer that has been used in the production of IPMCs by FFF 3D printing. In 2017, S. Trabia and co-workers reported that the use of Aquivion, a Nafion polymeric analogue possessing shorter side chains, led to better performance in terms of ionic conductivity (228 vs. 100 mS·cm^−1^), IEC (1.25 vs. 0.98 meq·g^−1^) and thermal conductivity (0.308 vs. 0.289 W·mK^−1^) as well as having a lower melting temperature (230 vs. 290 °C).

The Aquivion filament was produced by fusing precursor pellets and was extruded at 260 °C, with a speed rate of 10 mm s^−1^ and a bed at 180 °C. The printed Aquivion IPMC (Pr-Aq-IPMC) was compared to a traditionally made Nafion-based IPMC (N-IPMC) and showed four times larger displacements (over four acquisitions) even at 5 Hz, which is considered a fast input for these smart materials, and generated a higher blocking force than the N-IPMC (7.86 vs. 1.04 mN) [87], thus demonstrating that Aquivion is a valid option for IPMC membrane obtained via AM.

In the above contributions, it has been shown that it is possible to print objects with complex structures in Nafion and Aquivion, thus overcoming the processability problems of these materials. The imitation of this 3D printing technique for the creation of IPMC lies in the definition of the shapes of the objects when compared to other 3D printing techniques.

#### 2.2.2. IPMC Devices from DIW Methodology

The use of DIW to create IPMCs is still in its infancy. This technique might have a higher applicability than FDM because of the room temperature printing processability that opens up possibilities to print different feedstock polymers, and because it is possible to avoid the extra step of Nafion oxidation. Y. Wang and co-workers focused their research on 3D printing with Nafion-CNT membrane using this technique. In 2018, they published their first work using Nafion and single-walled carbon nanotubes (SWCNT) as the base components of the 3D printed membrane. They prepared a Nafion-based ink by mixing a commercial Nafion dispersion (DE520) with *N*,*N*-dimethylacetamide (DMAC) in a 1:4 *v*/*v* ratio and concentrating it to the right viscosity. The mixture was printed, giving a sandwich structure with five layers of SWCNTs (with a thickness of 100 μm) per side and, in the middle, 10 layers of Nafion (with a thickness of 200 μm). It was tested as a pressure sensor and showed a sensitivity about 5.95 mV·N^−1^ over eight acquisitions; the sensor response was linear with high accuracy in the measured range [88].

**Figure 5:**
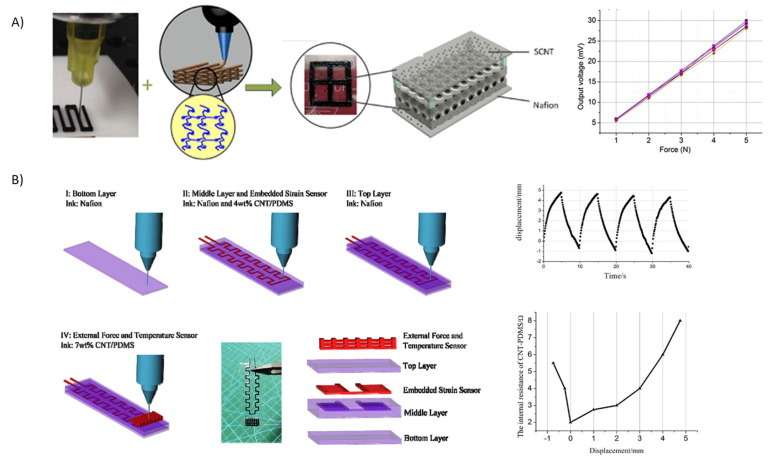
Nafion-based IPMC printed by DIW. Adapted from refs. [88,89]. For reference [88]: reproduced with permission © 2023 Elsevier Ltd., Amsterdam, The Netherlands. (**A**) DIW of a sandwich Nafion-SCNT IPMC and its sensitivity as pressure sensor; (**B**) (left) Preparation of a Nafion/CNT/PDMS IPMC; (right) Actuation and sensitivity characterization of this IPMC.

In 2021, the same group published a paper regarding the creation of an IPMC by using DIW. A Nafion-based ink was prepared using the procedure detailed before. To increase the material’s conductivity, they also separately prepared a CNT-polydimethylsiloxane (PDMS)-based ink by dispersing the CNTs in isopropyl alcohol (IPA) (1:50 wt%), subsequently adding the PDMS and removing the excess IPA by heating at 80 °C (Figure 5B). The IPMC/CNT-PDMS actuator was created via layer-by-layer deposition. It was composed of two layers (200 μm) of Nafion, molded with a speed of 15 mm·s^−1^ and an extrusion pressure of 40 kPa, embedding two further layers of 4 wt% CNT-PDMS, and extruded with 0.45 MPa at a speed of 10 mm·s^−1^. Finally, before platination, a last layer of Nafion, which was a CNT-PDMS 7%-based structure, was deposited. As shown in Figure 5B, the actuator demonstrated a displacement of nearly 6 mm (after the application of four successive stimuli) and sensitivity to applied forces between 2 and 3 mN [89].

In the above contributions, it has been shown that it is possible to print objects with complex and hollowed structures layered in Nafion and conducting materials such as carbon nanotubes. The challenge for future contributions in this field will be the preparation of new IPMCs in which the entire polymer membrane consists of a Nafion-conducting material composite.

#### 2.2.3. IPMC Devices from IP Methodology

**Figure 6:**
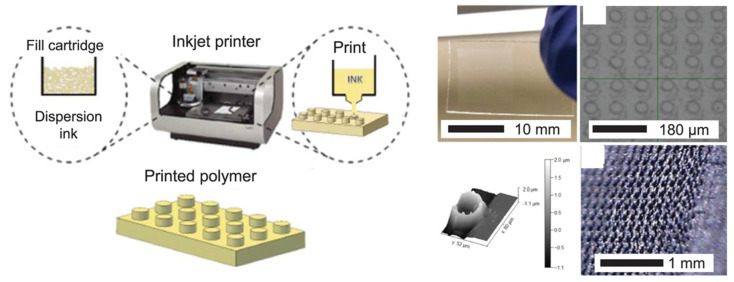
Nafion-based IPMC printed by IP technique. Adapted from Ref. [90].

The use of IP in this field is very recent and researchers (the M. Aureli group from the University of Nevada is especially active) are still trying to understand whether this technique could provide advantages over traditional techniques or FDM in the production of IPMCs. The Aureli group was able to produce several Nafion-based membranes with a hole pattern of 43–51 μm in diameter separated from each other by about 92 μm (Figure 6).

These membranes were shown to have 2.33 times the capacitance of a control membrane obtained from a commercial Nafion-N1117-based membrane (8.43 vs. 6.32 μF). The authors also tested the devices as pressure sensors and found an increase in the open circuit voltage as the applied pressure increased but no linear trend, as found in tests on membranes produced from commercial Nafion. The authors suggested that this behavior could be ascribed to fractures occurring within the material during the experiments [90].

The number of contributions published regarding the use of IP for the fabrication of IPMCs is still very limited, so it is difficult to highlight the strengths or weaknesses of the technique.

#### 2.2.4. Stereolithography (SLA)

To date, SLA has not attracted much research attention as regards the preparation of IPMC devices. The two papers dealing with IPMCs mention the use of this technique; they are limited to using SLA for the creation of substrates to which IPMCs synthesized from commercial membranes [91] are anchored, or substrates from which the membrane is then casted [92]. This printing technique could be used to obtain IPMCs with complex structures, as demonstrated using DLP (see below).

#### 2.2.5. Digital Light Processing (DLP)

The use of DLP for the production of IPMCs can be considered as the prominent innovation in the field, because it does not require Nafion or analogues and allows production to move towards the use of other anionic materials [93,94]. Engel and co-workers prepared a new IPMC using the DLP technique by using a resin composed of butyl acrylate and 2-acrylamido-2-methyl-1-propanesulfonic acid (AMP) as co monomers, 1,6-hexanediol acrylate as the crosslinker, and diphenyl (2,4,6-trimethylbenzoyl)phosphine oxide (TPO) as the photoinitiator, dissolved in DMSO. After curing, the membrane was coated with two layers of silver. The authors tested the ion exchange capacities with different counterions (Li^+^ and Mg^2+^) and the membrane showed a reduction of 10% in exchange capacity with respect to the Nafion-117-based reference. The result is encouraging given the lower number of sulfonic groups within the resin. Regarding charge mobility, the material loses 90% of its capacity by doubling the level of cross-linking from 5 to 10% when lithium ions are considered. The authors measured the bending angle of the actuator exposed to a sinusoidal waveform with a frequency of 10 MHz and amplitude of 3 V. At an equal cross-linking percentage, the actuator with lithium ion shows three times more displacement than the magnesium actuator, probably due to the larger size of the ion and lower concentration of charge carriers. By doubling the sulfone groups for the same cross-linking, the displacement increases (1.8 vs. 2.0 mm) for the lithium-based actuator; whereas, in the case of the magnesium actuator, the displacement was doubled (0.6 vs. 1.2 mm) [95].

Wang and co-workers focused their attention on a poly(acrylic acid-vinylimidazole) (PAAVim)-based membrane, a new example in the field of IPMCs, and tried to take advantage of the high ion exchange capacity [96] and easy synthesis of PAA. They printed different membranes, with increasing molar fractions of Vim, from 0 to 35 percent.

**Figure 7:**
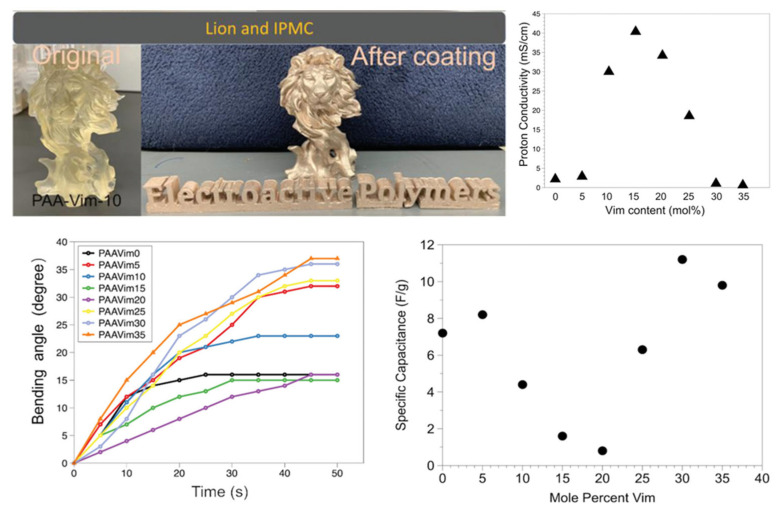
IPMC printed by DLP. Adapted with permission from Ref. [97].

As the molar fraction of Vim increases, the glass transition temperature (*T_g_*) and decomposition temperature (*T_d_*) increase because pVim has higher values than PAA: 144 vs. 79 °C (*T_g_*) and 370 vs 193 °C (*T_d_*), respectively. The same behavior does not occur with proton conductivity and stretchability; the optimum value is reached with a Vim mole fraction of 15%: 45 mS·cm^−1^ and 1227% of strain. Despite the higher proton conductivity, the membrane bending angle at 15 percent Vim is 2.5 times smaller than that at 30 percent Vim (14 vs. 37°), as is the specific capacitance (seven times lower, 1.6 vs. 11.2 F·*g*^−1^) (Figure 7) [97].

The DLP technique has the highest potential to create functional 3D-printed IPMCs, despite being constrained by the use of light-curable materials. A potential, foreseeable development will be IPMCs in which Nafion or Aquivion is encapsulated in a photoresin. In Table 2, we summarize advantages and disadvantages of the 3D printing techniques applied to the fabrication of IPMCs covered in this review.

## 3. Conclusions

This paper summarizes recent developments in the 3D printing of core ionomeric polymer material of IPMCs. Ionic-polymer metal composites hold great potential in the development of soft robotics, and the use of 3D printing techniques can enable actuators with complex geometries that would be difficult to produce using subtractive manufacturing techniques. Research in this area is very recent, and the results are encouraging. Photopolymerization-based SLA and DLP methodologies, which have recently been adopted for a wide range of materials [98,99,100,101,102], have not been as deeply investigated as material extrusion-based techniques such as FDM or DIW, but these additive manufacturing techniques may hold future potential for IPMC fabrication.

## Figures and Tables

**Table:** 

**3D Printing Method**	**Highlights**	**Weakness**	**Reference**
FDM	Low cost, fast printing time, commercially available filaments.	Low resolution of printed objects, not applicable to all polymers.	[25,26,27]
DIW	Low cost, easy printing process, wide range of materials.	Low resolution and low printing speed.	[28,29,30]
IP	Adaptable to large scale, small amount of ink and low amount of waste	Cost of ink, low printing speed.	[31,32,33]
SLA	High printing resolution	Long printing time, toxic monomers.	[34]
DLP	Short printing time, high resolution.	Toxic monomers	[3,35]

**Table 1:** Comparison of main advantages and weaknesses for IPMC membrane preparation methods.

Technique	Advantages	Drawbacks	Ref.
Solution Casting	Multifunctional layer preparation.	Cracks and bubbles formation	[66,67]
Hot Pressing	Cost effective and absence of boundary layer.	Set-up flexibility	[68]
3D Printing	Creation of complex shapes and high resolution.	Long printing time or use of toxic liquid resins	[35,69]

**Table 2:** Comparison of main advantages and drawbacks of IPMC membrane preparation by focusing the context exclusively on each 3D printing technique.

Technique	Advantages	Drawbacks	Ref.
Fused Deposition Modeling (FDM)	Low-cost materials and instruments	Low shape resolution	[82,83,84,85,86,87]
Direct Ink Writing (DIW)	Embedding conductive materials	Long time to optimize printing parameters	[88,89]
Inkjet Printing (IP)	High Resolution	Lower degree of applicability	[90]
Digital Light Processing (DLP)	High Resolution of complex structures	Worse performing membranes	[95,97]

## Data Availability

Not applicable.
